# Lipoprotein(a) and residual vascular risk in statin-treated patients with first acute ischemic stroke: A prospective cohort study

**DOI:** 10.3389/fneur.2022.1004264

**Published:** 2022-11-03

**Authors:** Lanjing Wang, Lijun Liu, Yanhong Zhao, Min Chu, Jijun Teng

**Affiliations:** ^1^Department of Neurology, The Affiliated Hospital of Qingdao University, Qingdao University, Qingdao, China; ^2^Department of Neurology, Minhang Hospital, Fudan University, Qingdao, China

**Keywords:** lipoprotein(a), stroke, statin, recurrence, vascular risk

## Abstract

**Objectives:**

Statins either barely affect or increase lipoprotein(a) [Lp(a)] levels. This study aimed to explore the factors correlated to the change of Lp(a) levels as well as the relationship between Lp(a) and the recurrent vascular events in statin-treated patients with first acute ischemic stroke (AIS).

**Methods:**

Patients who were admitted to the hospital with first AIS from October 2018 to September 2020 were eligible for inclusion. Correlation between the change of Lp(a) levels and potential influencing factors was assessed by linear regression analysis. Cox proportional regression models were used to estimate the association between Lp(a) and recurrent vascular events including AIS, transient ischemic attack, myocardial infarction and coronary revascularization.

**Results:**

In total, 303 patients, 69.6% males with mean age 64.26 ± 11.38 years, completed the follow-up. During the follow-up period, Lp(a) levels increased in 50.5% of statin-treated patients and the mean percent change of Lp(a) levels were 14.48% (95% CI 6.35–22.61%). Creatinine (β = 0.152, 95% CI 0.125–0.791, *P* = 0.007) and aspartate aminotransferase (AST) (β = 0.160, 95% CI 0.175–0.949, *P* = 0.005) were positively associated with the percent change of Lp(a) levels. During a median follow-up of 26 months, 66 (21.8%) patients had a recurrent vascular event. The median time period between AIS onset and vascular events recurrence was 9.5 months (IQR 2.0–16.3 months). The on-statin Lp(a) level ≥70 mg/dL (HR 2.539, 95% CI 1.076–5.990, *P* = 0.033) and the change of Lp(a) levels (HR 1.003, 95% CI 1.000–1.005, *P* = 0.033) were associated with the recurrent vascular events in statin-treated patients with first AIS. Furthermore, the on-statin Lp(a) levels ≥70 mg/dL (HR 3.612, 95% CI 1.018–12.815, *P* = 0.047) increased the risk of recurrent vascular events in patients with low-density lipoprotein cholesterol (LDL-C) levels < 1.8 mmol/L.

**Conclusions:**

Lp(a) levels increased in half of statin-treated patients with first AIS. Creatinine and AST were positively associated with the percent change of Lp(a) levels. Lp(a) is a determinant of residual vascular risk and the change of Lp(a) is positively associated with the risk of recurrent vascular events in these patients.

## Introduction

Hyperlipidemia is one of the most important causal risk factors for atherosclerotic cardiovascular disease (ASCVD) and stroke (1, 2). Treatment of hyperlipidemia is a vital aspect of the secondary prevention in these diseases (3). Statins can significantly reduce the low-density lipoprotein cholesterol (LDL-C) levels and are the most widely used lipid-lowering drugs (4). Even so, statin therapy has its limitations, such as increasing the risk of statin-associated myopathy and hepatotoxicity (5). Besides, statin therapy may increase lipoprotein(a) [Lp(a)] levels in patients with ASCVD (6).

Lp(a) is composed of a LDL-like particle in which apolipoprotein B100 is covalently linked by a disulfide bond to apolipoprotein(a) [apo(a)] (7). Regulated by the LPA gene encoding apo(a) and rarely affected by age, gender, or lifestyle, Lp(a) levels vary spectacularly (up to 1,000-fold) among individuals, and ~20% of the population have elevated Lp(a) levels (>30 mg/dL) (8–11). In the past long time, Lp(a) had been relatively neglected on account of no satisfactory therapeutic methods to reduce Lp(a) levels (12). However, there is ample evidence that elevated Lp(a) levels are significantly and independently associated with ASCVD and stroke, which may be attributes to the proatherogenic, proinflammatory, and potentially antifibrinolytic effects of Lp(a) (13–17). Moreover, the contribution of Lp(a) in cardiovascular disease (CVD) is not less than LDL-C (18). In recent years, the interest in Lp(a) has been reignited as a consequence of the evidence on its causality for CVD and the emergence of new targeted therapeutics including proprotein convertase subtilisin/kexin type 9 (PCSK9) inhibitors, Lp(a) apheresis (LA), and RNA-targeted therapies which dramatically lower Lp(a) levels (19, 20).

Existing studies suggested that statin-treated individuals had the higher recurrent cardiovascular risk compared with those who did not taking statins when LDL-C levels were similar (21). Besides, the recurrent cardiovascular risk was positively correlated with the on-statin Lp (a) levels (22). The residual risk for cardiovascular events in patients receiving statin therapy may be mainly associated with Lp(a) (23). By comparison, evidence on residual risk in statin-treated patients with acute ischemic stroke (AIS) is insufficient. In this study, we aimed to explore the factors related to the change of Lp(a) levels as well as the association between the change of Lp(a) levels and recurrence of vascular events in statin-treated patients with first AIS.

## Methods

### Participants enrollment and baseline measurements

The present study was a single-center, prospective, observational cohort study conducted at the Affiliated Hospital of Qingdao University. Ethical approval was obtained from the Ethics Committee of the Affiliated Hospital of Qingdao University and informed consent was obtained from all patient or their family members.

Patients admitted to the hospital within 7 days of symptom onset with first AIS from October 2018 to September 2020, who did not take lipid-lowering drugs before onset and agreed to participate this study were consecutively screened for inclusion. Those with other serious medical diseases including renal failure, hepatic failure, the late stage of malignant tumor or with a life expectancy < 24 months were excluded. The diagnoses of AIS were confirmed based on clinical manifestations and signs in combination with cranial computerized tomography (CT) and/or magnetic resonance imaging (MRI). Two experienced neurologists assessed the stroke severity at admission using the National Institutes of Health Stroke Scale (NIHSS) and analyzed the stroke etiology according to the Trial of Org 10,172 in Acute Stroke Treatment (TOAST) criteria (24).

The following data of all participants were collected: age, gender, history of hypertension, diabetes mellitus, atrial fibrillation (AF), coronary heart disease (CHD), smoking and drinking, intravenous thrombolysis, intracranial or extracranial vascular stenosis, laboratory tests [i.e., Lp(a), triglyceride (TG), total cholesterol (TC), low-density lipoprotein cholesterol (LDL-C), high-density lipoprotein cholesterol (HDL-C), fasting blood glucose (FBG), urea nitrogen, uric acid, creatinine, aspartate aminotransferase (AST), alanine aminotransferase (ALT), prothrombin time (PT), activated partial thromboplastin time (APTT) and D-Dimer]. All blood samples tested in the laboratory were obtained in fasting state within 24 h after admission. Lp(a) levels were measured using immunoturbidimetric method by Olympus 2,700 (Olympus, Japan). On the basis of previous literature, Lp(a) levels of all participants were categorized into 5 groups: < 15, 15–30 mg/dL, 30–50, 50–70, and ≥70 mg/dL groups (25).

### Follow-up and end points

The end points of this study included AIS, transient ischemic attack (TIA), myocardial infarction (MI) and coronary revascularization. Patients with recurrence of end points were followed up face-to-face when they were re-admitted. The follow-up time of these patients was calculated as the time from AIS onset to the end point events and their blood lipid levels were measured with the same detection method when end points occurred. In contrast, free-event patients, confirmed through telephone or face-to-face follow-up, were those who did not suffer from end points during a 2-year follow-up period and their blood lipid levels were assessed at the end of follow-up. The percent change of Lp(a) level was calculated as [the radio of follow-up Lp(a) level minus baseline Lp(a) level and baseline Lp(a) level]. Patients who lost to follow-up, declined to continue, discontinued statins or died due to conditions except end point events during follow-up were excluded from analysis.

### Statistical analysis

Baseline data were described by summary statistics. We compared data between the free-event group and the end point group. The quantitative data were indicated by mean and standard deviation or median and interquartile range and compared by using Student's *t*-test or Mann-Whitney U-test depending on type of data distribution. The qualitative data was indicated by frequency and percentage, and compared by using Chi-squared test.

Wilcoxon text was used to compare the difference between baseline and follow-up blood lipid levels. Correlation between baseline Lp(a) levels and follow-up Lp(a) levels were assessed by Spearman correlation analysis. Univariate linear regression analysis was used to screen the influencing factors of the percent change of Lp(a) levels. Variables with a *P*-value of < 0.10 in univariate analysis were subsequently entered into a multivariable linear regression analysis to determine the independent factors associated with the percent change of Lp(a) levels in statin-treated patients with first AIS. A two-tailed *P* < 0.05 was considered statistically significant.

Cumulative survival free of recurrent vascular events during follow-up were assessed using Kaplan–Meier survival analysis followed by the log rank test. Univariate and multivariate Cox regression analysis were used to determine the associations between Lp(a) and recurrent vascular events. The multivariable Cox regression analysis were adjusted for the possible confounders, i.e., age, gender, hypertension, diabetes mellitus, AF, CHD, LDL-C, HDL-C, TC and TG. Three models were set up. Model 1 adjusted for age, gender. Model 2 adjusted for model 1 plus history of hypertension, diabetes, AF, and CHD. Model 3 adjusted for model 2 plus LDL-C, HDL-C, TC and TG. Results were expressed as hazard ratio (HR) with the corresponding 95% confidence interval (CI). A two-tailed *P* < 0.05 was considered statistically significant. All statistical analysis in this study was performed with Statistical Package for the Social Sciences (SPSS) software version 26.0 (SPSS, Inc., Chicago, IL, USA).

## Results

### Baseline characteristics

**Figure 1:**
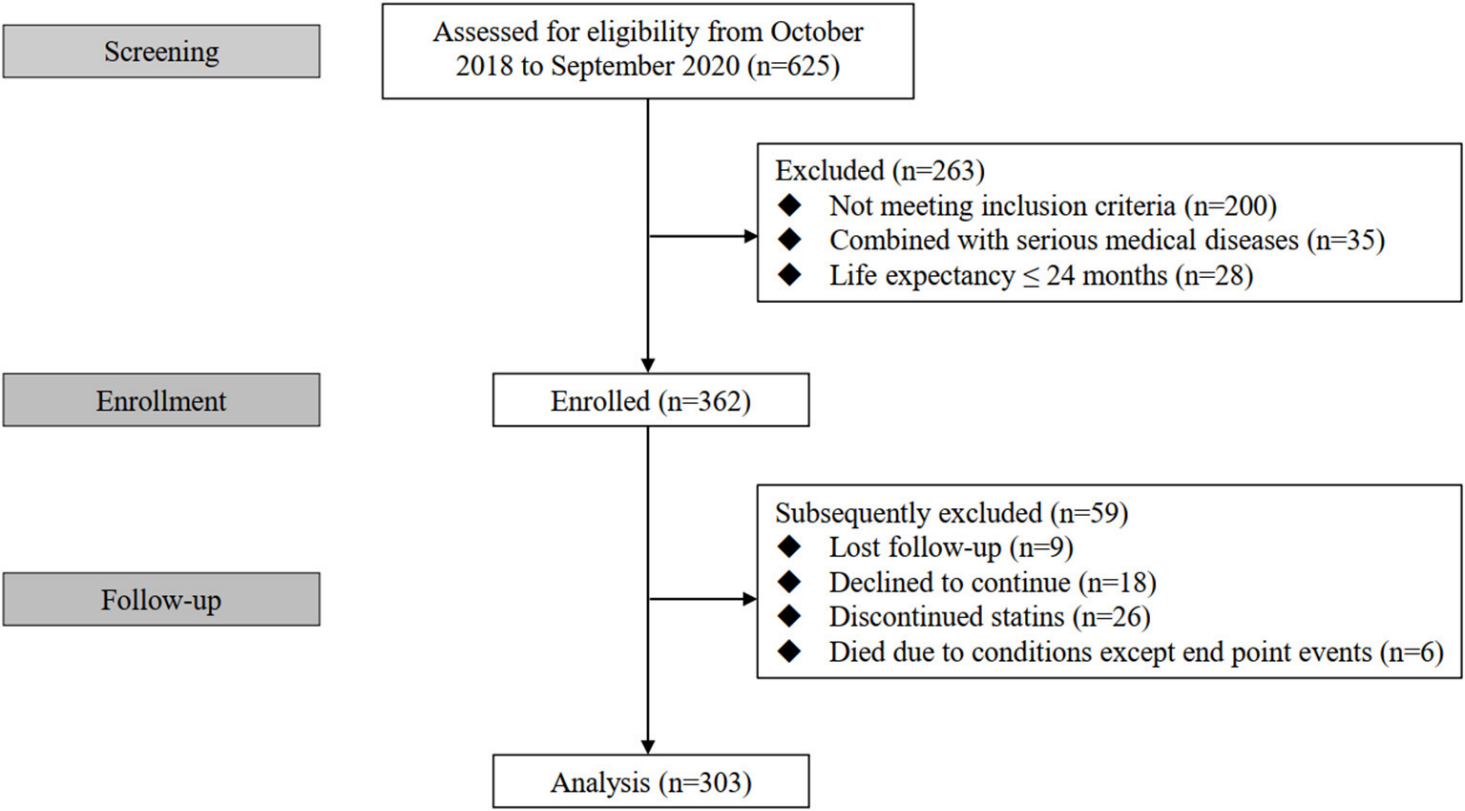
Flow diagram of the study.

Three hundred and sixty two eligible patients were initially enrolled in this study among whom 303 cases completed the follow-up and were analyzed finally (nine patients were lost to follow-up, 18 patients declined to continue, 26 patients discontinued statins and six patients died due to conditions except end point events during follow-up) (Figure 1). All enrolled patients received Atorvastatin (10–40mg) or Rosuvastatin (5–20mg).

Characteristics of the patients in the study cohort at baseline were presented in Table 1. Their mean age was 64.26 years and males accounted for 69.6%. With a median NIHSS of 4, 11.9% of patients received intravenous thrombolysis. The most common vascular risk factor in this study was hypertension (72.9%), followed by diabetes mellitus (37.6%). The etiological distribution according to the TOAST classification was large-artery atherosclerosis (LAA) 47.5%, cardioembolism (CE) 5.3%, small-artery occlusion (SAO) 27.4%, stroke of other determined etiology (SOE) 3.0% and stroke of undetermined etiology (SUE) 16.8%. The median baseline Lp(a) level was 18.90 mg/dL (IQR 11.20–31.00 mg/dL) and 79 (26.1%) patients had elevated Lp(a) levels (>30 mg/dL). In addition, 19 (6.3%), 43 (14.2%) and 23 (7.6%) patients had elevated TC levels (>6.2 mmol/L), TG levels (>2.3 mmol/L) and LDL-C levels (>4.1 mmol/L), respectively.

**Table 1:** Baseline and follow-up characteristics of patients in the study cohort.

	**Total (*n* = 303)**	**Free-event patients (*n* = 237)**	**Patients with end points (*n* = 66)**	***P***
**Baseline**				
Age, years	64.26 (11.38)	63.58 (11.41)	66.68 (11.01)	0.047
Gender (men), *n* (%)	211 (69.6)	160 (67.5)	51 (77.3)	0.127
Intravenous thrombolysis, *n* (%)	36 (11.9)	32 (13.5)	4 (6.1)	0.098
NIHSS at hospitalization, *n* (%)	4 (1–6)	4 (1–7)	3 (1–5)	0.233
Hypertension, *n* (%)	221 (72.9)	169 (71.3)	52 (78.8)	0.226
Diabetes mellitus, *n* (%)	114 (37.6)	88 (37.1)	26 (39.4)	0.737
AF, *n* (%)	36 (11.9)	28 (11.8)	8 (12.1)	0.946
CHD, *n* (%)	49 (16.2)	35 (14.8)	14 (21.2)	0.209
Smoking, *n* (%)	68 (22.4)	59 (24.9)	9 (13.6)	0.053
Alcoholism, *n* (%)	49 (16.2)	42 (17.7)	7 (10.6)	0.165
Intracranial or extracranial vascular stenosis, *n* (%)	157 (51.8)	119 (50.2)	38 (57.6)	0.290
TOAST classification, *n* (%)				0.178
LAA	144 (47.5)	106 (44.7)	38 (57.6)	0.064
CE	16 (5.3)	15 (6.3)	1 (1.5)	0.217
SAO	83 (27.4)	64 (27.0)	19 (28.8)	0.774
SOE	9 (3.0)	8 (3.4)	1 (1.5)	0.706
SUE	51 (16.8)	44 (18.6)	7 (10.6)	0.126
Baseline Lp(a), mg/dL	18.90 (11.20–31.00)	18.90 (11.20–30.45)	18.95 (11.77–36.30)	0.739
< 15	112 (37.0)	86 (36.3)	26 (39.4)	0.644
15– < 30	112 (37.0)	91 (38.4)	21 (31.8)	0.327
30– < 50	43 (14.2)	32 (13.5)	11 (16.7)	0.515
50– < 70	22 (7.3)	16 (6.8)	6 (9.1)	0.704
≥70	14 (4.6)	12 (5.1)	2 (3.0)	0.716
LDL-C, mmol/L	2.57 (1.99–3.36)	2.61 (2.04–3.36)	2.44 (1.86–3.43)	0.291
HDL-C, mmol/L	1.08 (0.90–1.25)	1.09 (0.91–1.25)	1.01 (0.89–1.23)	0.217
TG, mmol/L	1.26 (0.96–1.87)	1.26 (0.95–1.82)	1.26 (0.98–2.21)	0.448
TC, mmol/L	4.28 (3.38–5.17)	4.30 (3.42–5.20)	4.17 (3.24–5.20)	0.646
ALT, U/L	16.30 (12.30–25.85)	17.00 (12.30–26.00)	15.00 (12.00–24.00)	0.181
AST, U/L	18.00 (14.00–23.00)	18.00 (14.00–23.80)	17.00 (14.00–22.00)	0.481
Urea nitrogen, mmol/L	5.12 (4.22–6.36)	5.24 (4.24–6.38)	4.60 (3.93–6.19)	0.136
Uric acid, μmol/L	308.50 (253.65–367.30)	306.50 (257.10–365.00)	317.00 (245.50–384.00)	0.599
Creatinine, μmol/L	76.85 (63.00–92.23)	76.70 (63.60–92.00)	77.00 (60.50–94.50)	0.988
FBG, mmol/L	5.62 (4.93–7.29)	5.58 (4.89–7.12)	5.74 (5.04–7.57)	0.478
D-Dimer, ng/mL	290.00 (190.00–480.00)	290.00 (195.00–475.00)	275.00 (160.00–485.75)	0.599
PT, sec	10.50 (9.40–11.50)	10.50 (9.50–11.60)	10.00 (9.10–11.40)	0.066
APTT, sec	30.20 (27.20–32.60)	29.90 (27.80–32.80)	30.45 (27.33–32.43)	0.916
**Follow-up**				
On-statin Lp(a), mg/dL	19.00 (10.95–34.20)	18.40 (10.72–33.22)	22.05 (11.53–40.35)	0.149
< 15	11.7 (386)	96 (40.5)	21 (31.8)	0.200
15– < 30	95 (31.4)	74 (31.2)	21 (31.8)	0.927
30– < 50	48 (15.8)	35 (14.8)	13 (19.7)	0.332
50– < 70	21 (6.9)	18 (7.6)	3 (4.5)	0.556
≥70	22 (7.3)	14 (5.9)	8 (12.1)	0.146
On-statin LDL-C, mmol/L	1.73 (1.38–2.24)	1.74 (1.38–2.22)	1.70 (1.37–2.35)	0.930
On-statin HDL-C, mmol/L	1.10 (0.95–1.27)	1.10 (0.95–1.28)	1.07 (0.89–1.22)	0.176
On-statin TG, mmol/L	1.16 (0.88–1.57)	1.16 (0.90–1.56)	1.16 (0.86–1.65)	0.969
On-statin TC, mmol/L	3.30 (2.78–3.86)	3.30 (2.82–3.81)	3.27 (2.73–3.88)	0.894

Quantitative data are given as mean (standard deviation) or median (interquartile range) and qualitative data as number (n) and percentage (%). NIHSS, National Institutes of Health Stroke Scale; AF, Atrial fibrillation; CHD, Coronary heart disease; TOAST, Trial of Org 10,172 in Acute Stroke Treatment; LAA, large-artery atherosclerosis; CE, cardioembolism; SAO, small-artery occlusion; SOE, stroke of other determined etiology; SUE, stroke of undetermined etiology; Lp(a), lipoprotein(a); LDL-C, low-density lipoprotein cholesterol; HDL-C, high-density lipoprotein cholesterol; TG, triglyceride; TC, total cholesterol; FBG, fasting blood glucose; ALT, alanine aminotransferase; AST, aspartate aminotransferase; PT, prothrombin time; APTT, activated partial thromboplastin time.

### Follow-up blood lipid levels

**Figure 2:**
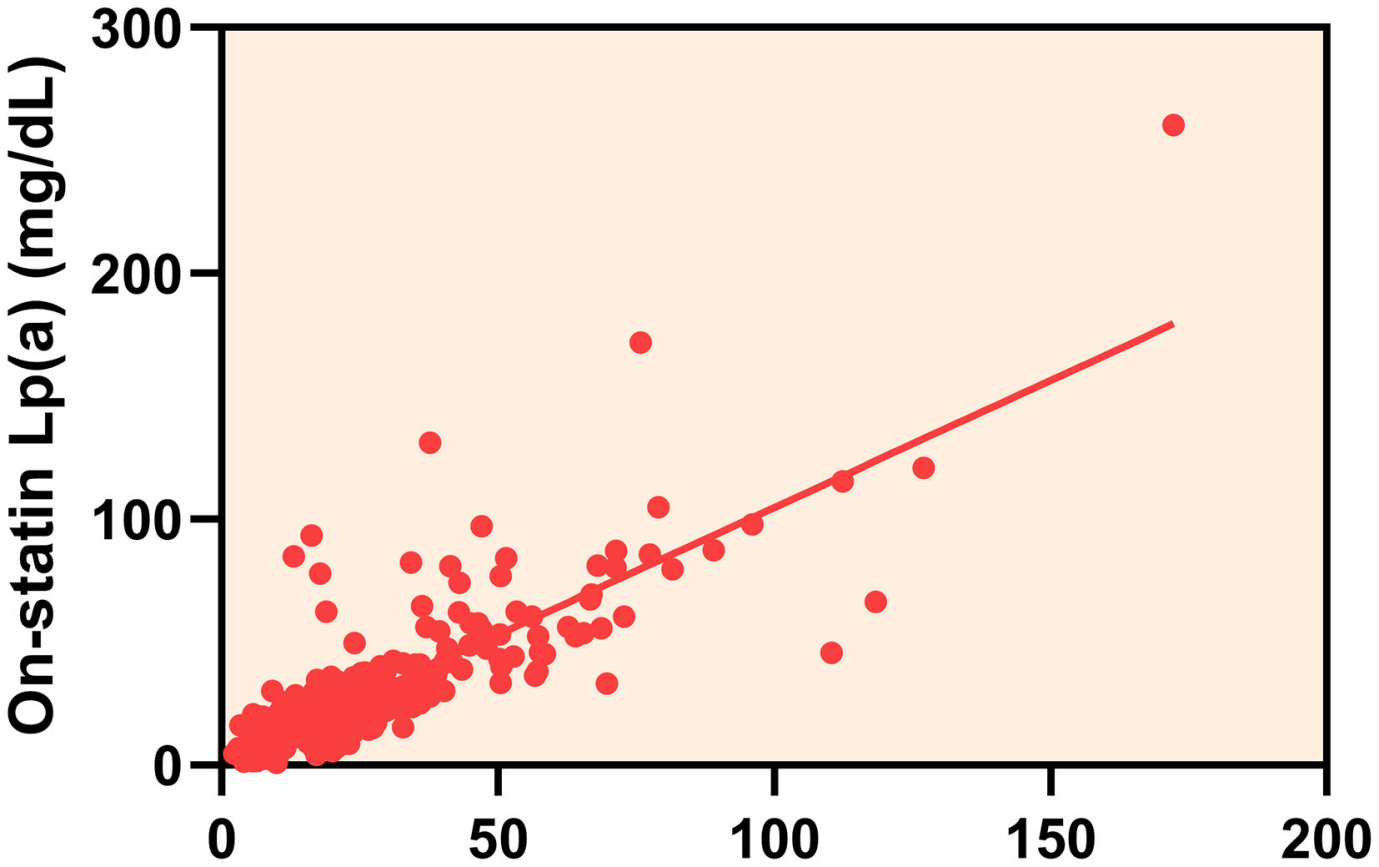
The correlation between baseline and on-statin lipoprotein(a) [Lp(a)] levels.

**Figure 3:**
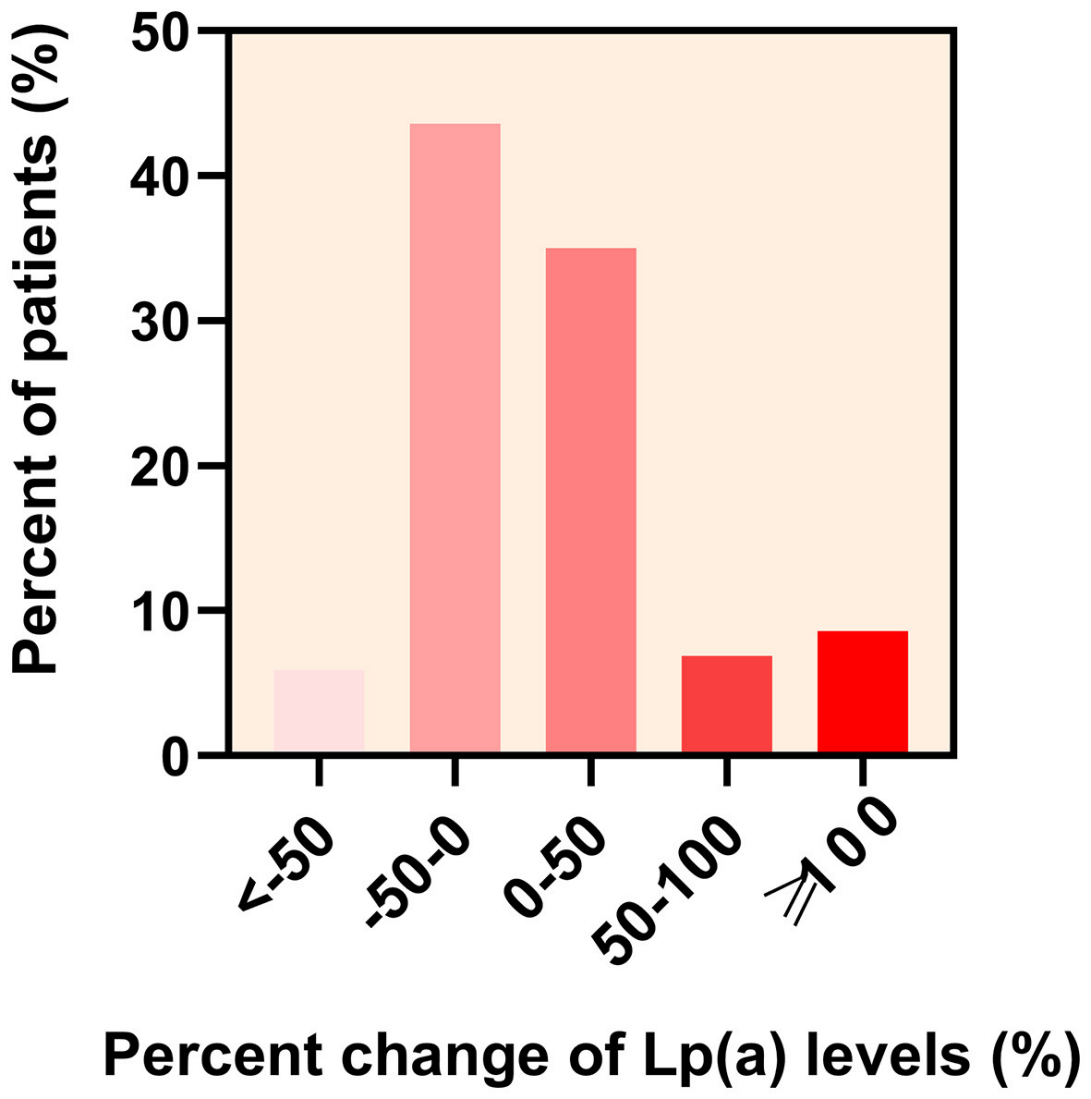
Distribution of the percent change of lipoprotein(a) [Lp(a)] levels in patients. The percent change of Lp(a) levels in statin-treated patients with first acute ischemic stroke at < −50,−50–0, 0–50, 50–100, and ≥100% was 5.9, 43.6, 35.0, 6.9, and 8.6%, respectively.

The overall median follow-up Lp(a) level was 19.00 mg/dL (IQR 10.95–34.20 mg/dL) and there was a strong correlation between baseline and follow-up Lp(a) levels (Spearman correlation rho: 0.824; *P* < 0.001) (Figure 2). During the follow-up period, 153 (50.5%) statin-treated patients' Lp(a) levels increased and the mean percent change of Lp(a) levels were 14.48% (95% CI 6.35–22.61%). The majority of the absolute percent change of Lp(a) levels were within 50% (Figure 3). There was a tendency for follow-up Lp(a) levels to be higher than baseline Lp(a) levels which could be obtained from the distribution of Lp(a) measurements in the two groups.

In addition, the LDL-C, TC and TG levels of patients decreased significantly after taking statins (2.57 vs. 1.73 mmol/L, *P* < 0.001; 4.28 vs. 3.30 mmol/L, *P* < 0.001; 1.26 vs. 1.16 mmol/L, *P* < 0.001). 162 (53.47%) statin-treated patients reached target LDL-C levels (< 1.8 mmol/L). The difference of HDL-C levels between baseline and follow-up were not significant (1.08 vs. 1.10 mmol/L, *P* = 0.192).

### Factors correlated to the change of Lp(a) levels

The results of univariate linear regression analysis showed that the *P*-values of creatinine (β = 0.168, 95% CI 0.169–0.844, *P* = 0.003), AST (β = 0.161, 95% CI 0.173–0.961, *P* = 0.005), TG (β = 0.156, 95% CI 1.997–12.210, *P* = 0.007), TC (β = 0.107, 95% CI−0.283–10.325, *P* = 0.063), FBG (β = 0.104, 95% CI−0.265–6.333, *P* = 0.071) and APTT (β = −0.099, 95% CI−2.944–0.192, *P* = 0.085) were < 0.10 (Table 2). Therefore, these factors were entered into a multivariable linear regression analysis, which indicated that creatinine (β = 0.152, 95% CI 0.125–0.791, *P* = 0.007) and AST (β = 0.160, 95% CI 0.175–0.949, *P* = 0.005) were independently associated with the percent change of Lp(a) levels in statin-treated patients with first AIS (Table 3).

**Table 2:** Univariate linear regression analysis on the association between baseline factors and the percent change of lipoprotein(a) levels.

	**B**	**95% CI**	**β**	***P***
Age	0.101	−0.617–0.818	0.016	0.783
Gender (men)	−1.349	−19.070–16.372	−0.009	0.881
Hypertension	−13.683	−31.959–4.593	−0.085	0.142
Diabetes mellitus	2.446	−14.374–19.265	0.016	0.775
AF	−7.055	−32.227–18.117	−0.032	0.582
CHD	−0.967	−23.099–21.165	−0.005	0.932
Stroke etiology (LAA)	5.796	−10.509–22.101	0.040	0.485
Intracranial or extracranial vascular stenosis	7.274	−9.014–23.562	0.051	0.380
Lp(a)	−0.296	−0.661–0.068	−0.092	0.111
LDL-C	−1.572	−9.688–6.544	−0.022	0.703
HDL-C	18.644	−8.377–45.664	0.078	0.176
TG	7.103	1.997–12.210	0.156	0.007
TC	5.021	−0.283–10.325	0.107	0.063
ALT	−0.124	−0.582–0.334	−0.031	0.595
AST	0.567	0.173–0.961	0.161	0.005
Urea nitrogen	0.529	−3.597–4.655	0.015	0.801
Uric acid	0.029	−0.062–0.121	0.036	0.531
Creatinine	0.506	0.169–0.844	0.168	0.003
FBG	3.034	−0.265–6.333	0.104	0.071
D-Dimer	−0.022	−0.012–0.007	−0.029	0.618
PT	−0.101	−6.837–4.634	−0.022	0.706
APTT	−1.376	−2.944–0.192	−0.099	0.085

CI, confidence interval; AF, Atrial fibrillation; CHD, Coronary heart disease; LAA, large-artery atherosclerosis; Lp(a), lipoprotein(a); LDL-C, low-density lipoprotein cholesterol; HDL-C, high-density lipoprotein cholesterol; TG, triglyceride; TC, total cholesterol; FBG, fasting blood glucose; ALT, alanine aminotransferase; AST, aspartate aminotransferase; PT, prothrombin time; APTT, activated partial thromboplastin time.

**Table 3:** Multivariable linear regression analysis on the association between baseline factors and the percent change of lipoprotein(a) levels.

	**B**	**95% CI**	**β**	***P***
Creatinine	0.458	0.125–0.791	0.152	0.007
AST	0.562	0.175–0.949	0.160	0.005
TG	5.246	−0.078–10.570	0.115	0.053
TC	2.393	−3.125–7.910	0.051	0.394
FBG	1.100	−2.327–4.526	0.038	0.528
APTT	−1.166	−2.697–0.365	−0.084	0.135

AST, aspartate aminotransferase; TG, triglyceride; TC, total cholesterol; FBG, fasting blood glucose; APTT, activated partial thromboplastin time.

### Lp(a) and end point events

During a median follow-up of 26 months, there were a total of 66 recurrent vascular events (61 AIS, 1 TIA, 2 MI and 2 coronary revascularizations). The median time period between AIS onset and vascular events recurrence was 9.5 months (IQR 2.0–16.3 months). Individuals with recurrent vascular events were older than those without events (63.58 vs. 66.68 years, *P* = 0.047). No significant differences were observed in other baseline characteristics (Table 1).

**Figure 4:**
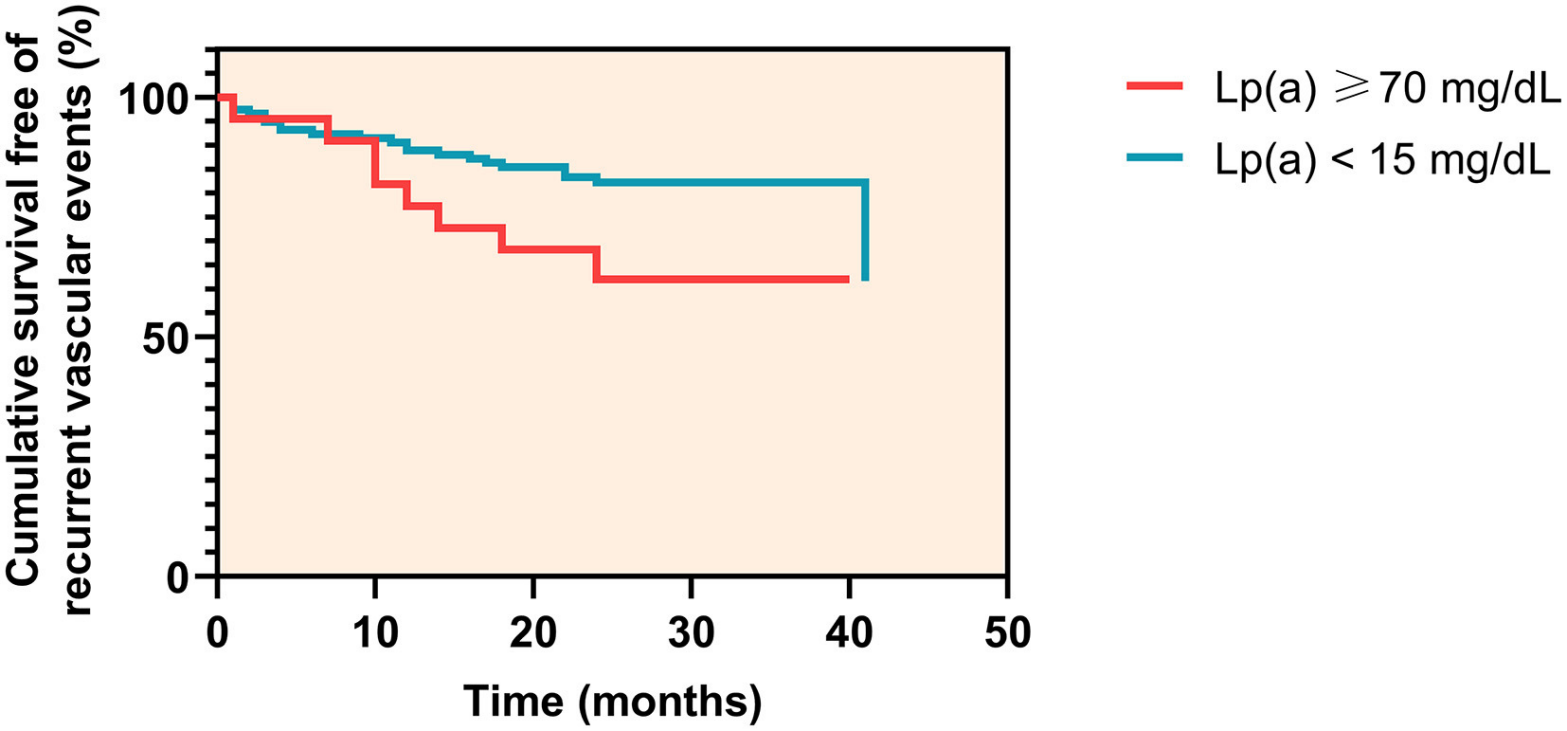
Kaplan-Meier curves for cumulative survival free of recurrent vascular events.

Among patients with Lp(a) levels < 15 mg/dL, 21 of 117 patients (17.9%) experienced a recurrent vascular event at a median time of 11 months (IQR 7.8–17.0 months), whereas in patients with Lp(a) levels ≥ 70 mg/dL, 8 of 22 patients (36.4%) experienced an event at a median time of 11 months (IQR 3.0–17.5 months) (log rank *P* = 0.037) (Figure 4).

The association between baseline Lp(a) levels and recurrent vascular events was not significant in univariate or multivariate Cox regression analysis. On-statin Lp(a) level ≥70 mg/dL significantly increased the risk for recurrent vascular events after adjustment for relevant confounding factors (HR 2.539, 95% CI 1.076–5.990, *P* = 0.033). The increased percent change of Lp(a) levels tended to increase the risk for recurrent vascular events (HR 1.003, 95% CI 1.000–1.005, *P* = 0.033) (Table 4). In patients with LDL-C levels < 1.8 mmol/L, on-statin Lp(a) levels ≥70 mg/dL also increased the risk for recurrent vascular events after adjustment for relevant confounding factors (HR 3.612, 95% CI 1.018–12.815, *P* = 0.047) (Table 5). In multivariate Cox regression analysis, age was not independently associated with recurrent vascular events.

**Table 4:** Multivariate Cox regression analysis on the association between lipoprotein(a) levels and recurrent vascular events.

	**Baseline Lp(a)**			**On-statin Lp(a)**			**The percent change of Lp(a)**		
	**HR**	**95% CI**	***P***	**HR**	**95% CI**	***P***	**HR**	**95% CI**	***P***
Unadjusted							1.003	1.001–1.005	0.016
15– < 30	0.836	0.470–1.487	0.542	1.376	0.749–2.528	0.304			
30– < 50	1.225	0.604–2.486	0.573	1.790	0.891–3.598	0.102			
50– < 70	1.227	0.504–2.987	0.652	0.823	0.245–2.765	0.753			
≥70	0.641	0.152–2.703	0.544	2.322	1.025–5.263	0.044			
Model 1							1.003	1.001–1.005	0.014
15– < 30	0.850	0.478–1.513	0.581	1.480	0.803–2.728	0.208			
30– < 50	1.326	0.651–2.700	0.437	1.741	0.866–3.502	0.120			
50– < 70	1.191	0.489–2.900	0.701	0.841	0.250–2.833	0.780			
≥70	0.626	0.148–2.641	0.523	2.398	1.057–5.443	0.036			
Model 2							1.003	1.001–1.005	0.009
15– < 30	0.867	0.486–1.546	0.629	1.503	0.814–2.776	0.193			
30– < 50	1.354	0.663–2.765	0.405	1.758	0.873–3.542	0.114			
50– < 70	1.202	0.493–2.929	0.685	0.868	0.256–2.939	0.820			
≥70	0.577	0.135–2.476	0.460	2.403	1.039–5.558	0.040			
Model 3							1.003	1.000–1.005	0.033
15– < 30	0.869	0.483–1.565	0.640	1.533	0.826–2.846	0.176			
30– < 50	1.375	0.666–2.838	0.389	1.730	0.852–3.515	0.130			
50– < 70	1.194	0.471–3.027	0.709	0.912	0.265–3.137	0.884			
≥70	0.614	0.141–2.675	0.516	2.539	1.076–5.990	0.033			

Model 1: adjusted for age, gender. Model 2: adjusted for model 1 plus history of hypertension, diabetes, atrial fibrillation, and coronary heart disease. Model 3: adjusted for model 2 plus LDL-C, HDL-C, TC and TG [baseline Lp(a) adjusted for baseline blood lipid levels; on-statin Lp(a) adjusted for on-statin blood lipid levels; the change of Lp(a) adjusted for the change of blood lipid levels].

**Table 5:** Multivariate Cox regression analysis on the association between on-statin lipoprotein(a) levels and recurrent vascular events in patients with LDL-C levels < 1.8 mmol/L.

	**HR**	**95% CI**	***P***
**Unadjusted**			
15– < 30	1.461	0.654–3.263	0.355
30– < 50	2.351	0.945–5.849	0.066
50– < 70	0.766	0.099–5.937	0.766
≥70	3.910	1.243–12.300	0.020
**Model 1**			
15– < 30	1.547	0.686–3.489	0.355
30– < 50	2.318	0.930–5.799	0.071
50– < 70	0.835	0.106–6.592	0.864
≥70	3.849	1.219–12.150	0.022
**Model 2**			
15– < 30	1.510	0.665–3.426	0.193
30– < 50	2.512	1.001–6.303	0.051
50– < 70	0.821	0.103–6.556	0.852
≥70	3.708	1.097–12.536	0.035
**Model 3**			
15– < 30	1.423	0.609–3.326	0.415
30– < 50	2.419	0.941–6.219	0.067
50– < 70	0.765	0.094–6.237	0.803
≥70	3.612	1.018–12.815	0.047

Model 1: adjusted for age, gender. Model 2: adjusted for model 1 plus history of hypertension, diabetes, atrial fibrillation, and coronary heart disease. Model 3: adjusted for model 2 plus on-statin LDL-C, HDL-C, TC and TG.

## Discussion

To the best of our knowledge, this is the first study investigating the association between the change of Lp(a) with recurrent vascular events in a cohort of statin-treated patients with first AIS. Our results indicated that Lp(a) levels increased in half of statin-treated patients and the on-statin Lp(a) level ≥70 mg/d instead of the baseline Lp(a) level at admission was significantly associated with the recurrent vascular events. The relationship exists even the LDL-C levels are very low, suggesting that patients had residual vascular risk even while taking statins and Lp(a) is a determinant of the residual vascular risk. Moreover, the change of Lp(a) levels was positively associated with the risk of recurrent vascular events.

Statin therapy has minimal effect on Lp(a) even increases Lp(a) levels, which may be partly attributed to that LDL receptor does not play a major role in the clearance of Lp(a) (26). A meta-analysis showed that statins increase Lp(a) levels by 8.5–19.6% from baseline (6). In line with this study, we observed a 14.48% increasement in Lp(a) levels from baseline during statin therapy. Previous studies established that Lp(a) levels of individuals with low molecular weight apo (a) ( ≤ 22 kringle IV repeats) phenotype or baseline Lp(a) levels ≥70 nmol/L (≈34 mg/dL) were more likely to increase after taking statins (27, 28). And high-intensity statin therapy was more remarkably associated with increased Lp(a) levels in patients with CVD (29). Based on this situation, it is extremely necessary to explore which factors are associated with the change of Lp (a) levels in statin-treated AIS patients and whether the change of Lp (a) is associated with the recurrence risk for vascular events in these patients.

The underlying mechanisms by which statins affect circulating Lp(a) levels remain unclear. Statins elevate the expression of LPA mRNA as well as the synthesis and secretion of apo(a) protein in HepG2 cells, which may result in the increase of Lp(a) levels (6). Moreover, statins activate the expression of PCSK9 genes and increase PCSK9 levels, which then enhance Lp(a) production (30–32). Deficiently, there are few studies on the factors associated with the change of Lp(a) in statin-treated patients. The existing literature have provided some evidence that non-genetic factors can affect Lp(a) levels to a limited extent, such as kidney impairment, liver disease, inflammation, weight reduction or high saturated fat intake (33–38). In our study, we discovered that creatinine and AST were positively correlated with the change of Lp(a) levels despite these two factors can only explain the change to a small extent. The circulating Lp(a) levels are determined by the velocity of synthesis (39). The liver is responsible for the synthesis and catabolism of Lp(a) (40, 41). Therefore, liver dysfunction impairs Lp(a) metabolism (42). Available researches have validated that severe chronic liver diseases can lead to the decrease of Lp(a) synthesis so that the circulating Lp(a) levels can be decreased (36, 43). However, Lp(a) levels may be increased in some patients with hepatocarcinoma (44). Further studies are needed to validate the relationship between liver function and Lp(a) levels. Kidney may be involved in Lp(a) catabolism and the clearance of Lp(a) from circulation (45, 46). Therefore, renal impairment may block Lp(a) catabolism and result in the increase of circulating Lp(a) levels (47). Trinder et al. (28) discovered that there was a weak negative correlation between the percent change of Lp(a) and the estimated glomerular filtration rate. Large sample trials are warranted to investigate the factors associated with the influences of statins on Lp(a) levels.

Elevated Lp(a) levels are significantly and independently associated with the risk of ASCVD and Lp(a) has been confirmed as a momentous determinant of residual risk for cardiovascular events in statin-treated patients (13, 14, 23). But so far, few studies have investigated the residual vascular risks for AIS patients with elevated Lp(a) levels. Lange et al. (48) firstly reported that elevated Lp(a) levels contributed to the risk of recurrent vascular events after the first ischemic stroke in a 12-month follow-up study. Hong et al. (49) discovered that elevated Lp(a) levels were associated with early stroke recurrence in patients with AIS. The positive association between Lp(a) and the recurrence risk of cerebrovascular events was found in patients who were either < 60 years or had evident LAA stroke etiology (50). However, there is still a lack of studies on the relationship between the on-statin Lp(a) levels or the change of Lp(a) levels and the recurrence risk of vascular events in patients with AIS in a secondary prevention setting. Nestel et al. (51) claimed that increased Lp(a) levels after using statins 1 year were associated with future CVD events in patients with CHD. A latest study assessed the relationship between change of Lp(a) and risk of coronary artery disease and found the two mentioned above are not correlated (28). In this study, we discovered that the risk for recurrent vascular events is significantly higher in the patients whose on-statin Lp(a) levels ≥ 70 mg/dL and the change of Lp(a) was independently associated with the risk for recurrent vascular events in statin-treated patients with AIS, even in patients with LDL-C lower than 1.8 mmol/L. Furthermore, the risk associated with elevated Lp(a) increased in the context of very low LDL-C levels. Therefore, Lp(a) is a residual risk determinant in statin-treated patients with AIS.

The association between Lp(a) and residual risk of ASCVD makes Lp(a) a novel therapeutic target in ASCVD. Ongoing clinical trials provided some promising results that PCSK9 inhibitors, LA, and RNA-targeted therapies may be available Lp(a) lowering therapies (20). Existing studies have confirmed that lowering Lp(a) level by 80 mg/dL might reduce the risk of CHD by approximately 18 to 20% (52). A study reported that reducing Lp (a) level by 63% with LA achieved a 94% reduction in the major adverse cardiovascular events over a mean treatment period of 4 years (53). RNA-targeted therapies such as pelacarsen, olpasiran, and SLN360 which are currently investigated in trials have shown to lower Lp(a) levels by 90% (54). Decrease of Lp(a) levels by 50 mg/dL in 5 years in individuals with preexisting CVD may reduce the recurrence risk of CVD by 20% (55). The clinical benefits may be directly proportional to the absolute decrease of Lp (a) levels (52). Therefore, patients with higher baseline Lp(a) levels had greater reductions in the incidence of CVD events receiving Lp(a) lowering therapy (52, 56). Whether reducing Lp(a) levels in patients preexisting ischemic stroke can reduce the risk of recurrent vascular events deserves further study. And we should consider both patients' Lp(a) levels and their absolute risk of having a vascular event to determine whether they were likely to benefit from Lp(a)-lowering therapy (57).

Our study has some limitations. First, our study was a single center study, therefore, the sample size was relatively small. Second, a part of free-event patients was followed up by telephone, which might miss some end point events, especially TIA. Third, dietary habits and physical activities in the patients were not taken into account which might alter results of the study. In addition, it could not be ignored that the times of reaching the end point were different in patients, the measurement times of on-statin Lp(a) levels were different, so the total durations of statin use were different, which might cause certain errors in the results. The relationship between the change of Lp(a) and the recurrence of vascular events still needs to be confirmed by large-scale prospective studies. This study may provide evidence for the application of Lp(a) lowering therapy in AIS patients with elevated Lp(a) levels. Further prospective studies are warranted to evaluate the effect of Lp(a) lowering therapy on the recurrence of vascular events for patients with elevated Lp(a) levels and to identify those might benefit from Lp(a) lowering therapy.

## Conclusions

In the patients receiving statin therapy after first AIS, creatinine and AST were positively associated with the percent change of Lp(a) levels. Lp(a) is a determinant of residual vascular risk and the change of Lp(a) is positively associated with the risk of recurrent vascular events. For AIS patients with elevated Lp(a) levels, Lp(a) lowering therapy may be desirable. With the emergence of new lipid-lowering drugs, drugs that reduce LDL-C levels and do not increase Lp(a) levels can be considered in future treatment.

## Data availability statement

The raw data supporting the conclusions of this article will be made available by the authors, without undue reservation.

## Ethics statement

Written informed consent was obtained from the individual(s) for the publication of any potentially identifiable images or data included in this article.

## Author contributions

Study conception and design: JT. Acquisition of data: LW, YZ, and MC. Statistical analysis and drafting the manuscript: LW. Critical revision of the article for important intellectual content: LL and JT. All authors contributed to the article and approved the submitted version.

## Conflict of interest

The authors declare that the research was conducted in the absence of any commercial or financial relationships that could be construed as a potential conflict of interest.

## Publisher's note

All claims expressed in this article are solely those of the authors and do not necessarily represent those of their affiliated organizations, or those of the publisher, the editors and the reviewers. Any product that may be evaluated in this article, or claim that may be made by its manufacturer, is not guaranteed or endorsed by the publisher.

## References

[1] Hussain I Patni N Garg A. Lipodystrophies, dyslipidaemias and atherosclerotic cardiovascular disease. Pathology. (2019) 51:202–12. 10.1016/j.pathol.2018.11.004 30595509 PMC6402807

[2] Alloubani A Nimer R Samara R. Relationship between hyperlipidemia, cardiovascular disease and stroke: a systematic review. Curr Cardiol Rev. (2021) 17:e051121189015. 10.2174/1573403X16999201210200342 33305711 PMC8950504

[3] Tsai H Kim J Jouvent E Gurol M. Updates on prevention of hemorrhagic and lacunar strokes. J Stroke. (2018) 20:167–79. 10.5853/jos.2018.00787 29886717 PMC6007298

[4] Reith C Armitage J. Management of residual risk after statin therapy. Atherosclerosis. (2016) 245:161–70. 10.1016/j.atherosclerosis.2015.12.018 26722833

[5] Bellosta S Corsini A. Statin drug interactions and related adverse reactions: an update. Expert Opin Drug Saf. (2018) 17:25–37. 10.1080/14740338.2018.1394455 29058944

[6] Tsimikas S Gordts P Nora C Yeang C Witztum J. Statin therapy increases lipoprotein(a) levels. Eur Heart J. (2020) 41:2275–84. 10.1093/eurheartj/ehz310 31111151

[7] Schmidt K Noureen A Kronenberg F Utermann G. Structure, function, and genetics of lipoprotein (a). J Lipid Res. (2016) 57:1339–59. 10.1194/jlr.R067314 27074913 PMC4959873

[8] Trinder M Uddin M Finneran P Aragam K Natarajan P. Clinical utility of lipoprotein(a) and LPA genetic risk score in risk prediction of incident atherosclerotic cardiovascular disease. JAMA Cardiol. (2020) 6:1–9. 10.1001/jamacardio.2020.5398 33021622 PMC7539232

[9] Kronenberg F. Human genetics and the causal role of lipoprotein(a) for various diseases. Cardiovasc Drug Ther. (2016) 30:87–100. 10.1007/s10557-016-6648-3 26896185 PMC4789197

[10] Durlach V Bonnefont-Rousselot D Boccara F Varret M Di-Filippo Charcosset M Cariou B. Lipoprotein(a): pathophysiology, measurement, indication and treatment in cardiovascular disease. a consensus statement from the nouvelle société francophone d'athérosclérose (NSFA). Arch Cardiovasc Dis. (2021) 114:828–47. 10.1016/j.acvd.2021.10.009 34840125

[11] Swerdlow D Rider D Yavari A Wikström Lindholm M Campion G Nissen S. Treatment and prevention of lipoprotein(a)-mediated cardiovascular disease: the emerging potential of RNA interference therapeutics. Cardiovasc Res. (2022) 118:1218–31. 10.1093/cvr/cvab100 33769464 PMC8953457

[12] Tada H Takamura M Kawashiri M. Lipoprotein(a) as an old and new causal risk factor of atherosclerotic cardiovascular disease. J Atheroscler Thromb. (2019) 26:583–91. 10.5551/jat.RV17034 31061262 PMC6629747

[13] Vavuranakis M Jones S Cardoso R Gerstenblith G Leucker T. The role of Lipoprotein(a) in cardiovascular disease: current concepts and future perspectives. Hellenic J Cardiol. (2020) 61:398–403. 10.1016/j.hjc.2020.09.016 33039574 PMC8643135

[14] Mehta A Vasquez N Ayers C Patel J Hooda A Khera A. Independent association of lipoprotein(a) and coronary artery calcification with atherosclerotic cardiovascular risk. J Am Coll Cardiol. (2022) 79:757–68. 10.1016/j.jacc.2021.11.058 35210030 PMC10966924

[15] Arora P Kalra R Callas P Alexander K Zakai N Wadley V. Lipoprotein(a) and risk of ischemic stroke in the regards study. Arterioscler Thromb Vasc Biol. (2019) 39:810–8. 10.1161/ATVBAHA.118.311857 30786745 PMC6511401

[16] Tsimikas S. A test in context: lipoprotein(a): diagnosis, prognosis, controversies, and emerging therapies. J Am Coll Cardiol. (2017) 69:692–711. 10.1016/j.jacc.2016.11.042 28183512

[17] Tziomalos K Athyros V Wierzbicki A Mikhailidis D. Lipoprotein a: where are we now? Curr Opin Cardiol. (2009) 24:351–7. 10.1097/HCO.0b013e32832ac21a 19417640

[18] Langsted A Nordestgaard B. Lipoprotein(a): is it more, less or equal to LDL as a causal factor for cardiovascular disease and mortality? Curr Opin Lipidol. (2020) 31:125–31. 10.1097/MOL.0000000000000681 32304380

[19] Tokgozoglu L Orringer C Ginsberg H Catapano A. The year in cardiovascular medicine 2021: dyslipidaemia. Eur Heart J. (2022) 43:807–17. 10.1093/eurheartj/ehab875 34974612

[20] Kosmas C Sourlas A Mallarkey G Silverio D Ynoa D Montan P. Therapeutic management of hyperlipoproteinemia (a). Drugs Context. (2019) 8:212609. 10.7573/dic.212609 31555339 PMC6752750

[21] Lieb W Enserro D Larson M Vasan R. Residual cardiovascular risk in individuals on lipid-lowering treatment: quantifying absolute and relative risk in the community. Open Heart. (2018) 5:e000722. 10.1136/openhrt-2017-000722 29387429 PMC5786911

[22] Willeit P Ridker P Nestel P Simes J Tonkin A Pedersen T. Baseline and on-statin treatment lipoprotein(a) levels for prediction of cardiovascular events: individual patient-data meta-analysis of statin outcome trials. Lancet. (2018) 392:1311–20. 10.1016/S0140-6736(18)31652-0 30293769

[23] Khera A Everett B Caulfield M Hantash F Wohlgemuth J Ridker P. Lipoprotein(a) concentrations, rosuvastatin therapy, and residual vascular risk: an analysis from the JUPITER Trial (justification for the use of statins in prevention: an intervention trial evaluating rosuvastatin). Circulation. (2014) 129:635–42. 10.1161/CIRCULATIONAHA.113.004406 24243886 PMC3946056

[24] Adams H Bendixen B Kappelle L Biller J Love B Gordon D. Classification of subtype of acute ischemic stroke. definitions for use in a multicenter clinical trial. TOAST. Trial of Org 10172 in acute stroke treatment. Stroke. (1993) 24:35–41. 10.1161/01.STR.24.1.35 7678184

[25] Wong N Zhao Y Sung J Browne A. Relation of first and total recurrent atherosclerotic cardiovascular disease events to increased lipoprotein(a) levels among statin treated adults with cardiovascular disease. Am J Cardiol. (2021) 145:12–7. 10.1016/j.amjcard.2020.12.075 33454339 PMC8005472

[26] Saleheen D Haycock P Zhao W Rasheed A Taleb A Imran A. Apolipoprotein(a) isoform size, lipoprotein(a) concentration, and coronary artery disease: a mendelian randomisation analysis. Lancet Diabetes Endocrinol. (2017) 5:524–33. 10.1016/S2213-8587(17)30088-8 28408323 PMC5483508

[27] Yahya R Berk K Verhoeven A Bos S van der Zee L Touw J. Statin treatment increases lipoprotein(a) levels in subjects with low molecular weight apolipoprotein(a) phenotype. Atherosclerosis. (2019) 289:201–5. 10.1016/j.atherosclerosis.2019.07.001 31327478

[28] Trinder M Paruchuri K Haidermota S Bernardo R Zekavat S Gilliland T. Repeat measures of lipoprotein(a) molar concentration and cardiovascular risk. J Am Coll Cardiol. (2022) 79:617–28. 10.1016/j.jacc.2021.11.055 35177190 PMC8863206

[29] de Boer L Oorthuys A Wiegman A Langendam M Kroon J Spijker R. Statin therapy and lipoprotein(a) levels: a systematic review and meta-analysis. Eur J Prev Cardiol. (2021). 10.1016/j.atherosclerosis.2022.06.202 34849724

[30] Guo Y Liu J Xu R Zhu C Wu N Jiang L. Short-term impact of low-dose atorvastatin on serum proprotein convertase subtilisin/kexin type 9. Clin Drug Investig. (2013) 33:877–83. 10.1007/s40261-013-0129-2 24114461

[31] Awan Z Seidah N MacFadyen J Benjannet S Chasman D Ridker P. Rosuvastatin, proprotein convertase subtilisin/kexin type 9 concentrations, and LDL cholesterol response: the JUPITER trial. Clin Chem. (2012) 58:183–9. 10.1373/clinchem.2011.172932 22065156

[32] Villard E Thedrez A Blankenstein J Croyal M Tran T Poirier B. PCSK9 modulates the secretion but not the cellular uptake of lipoprotein(a) ex vivo: an effect blunted by alirocumab. JACC Basic Transl Sci. (2016) 1:419–27. 10.1016/j.jacbts.2016.06.006 29308438 PMC5753417

[33] Faghihnia N Tsimikas S Miller E Witztum J Krauss R. Changes in lipoprotein(a), oxidized phospholipids, and LDL subclasses with a low-fat high-carbohydrate diet. J Lipid Res. (2010) 51:3324–30. 10.1194/jlr.M005769 20713651 PMC2952573

[34] Kronenberg F Kuen E Ritz E Junker R König P Kraatz G. Lipoprotein(a) serum concentrations and apolipoprotein(a) phenotypes in mild and moderate renal failure. J Am Soc Nephrol. (2000) 11:105–15. 10.1681/ASN.V111105 10616846

[35] Lin J Reilly M Terembula K Wilson F. Plasma lipoprotein(a) levels are associated with mild renal impairment in type 2 diabetics independent of albuminuria. PLoS ONE. (2014) 9:e114397. 10.1371/journal.pone.0114397 25490096 PMC4260843

[36] Meroni M Longo M Lombardi R Paolini E Macchi C Corsini A. Low lipoprotein(a) levels predict hepatic fibrosis in patients with nonalcoholic fatty liver disease. Hepatol Commun. (2022) 6:535–49. 10.1002/hep4.1830 34677008 PMC8870034

[37] Reyes-Soffer G Westerterp M. Beyond lipoprotein(a) plasma measurements: lipoprotein(a) and inflammation. Pharmacol Res. (2021) 169:105689. 10.1016/j.phrs.2021.105689 34033878 PMC9247870

[38] Brandstätter A Lingenhel A Zwiauer K Strobl W Kronenberg F. Decrease of Lp(a) during weight reduction in obese children is modified by the apo(a) kringle-IV copy number variation. Int J Obes. (2009) 33:1136–42. 10.1038/ijo.2009.144 19636317

[39] Krempler F Kostner G Bolzano K Sandhofer F. Turnover of lipoprotein (a) in man. J Clin Invest. (1980) 65:1483–90. 10.1172/JCI109813 7410552 PMC371487

[40] Cain W Millar J Himebauch A Tietge U Maugeais C Usher D. Lipoprotein [a] is cleared from the plasma primarily by the liver in a process mediated by apolipoprotein [a]. J Lipid Res. (2005) 46:2681–91. 10.1194/jlr.M500249-JLR200 16150825

[41] Kronenberg F Utermann G. Lipoprotein(a): resurrected by genetics. J Intern Med. (2013) 273:6–30. 10.1111/j.1365-2796.2012.02592.x 22998429

[42] Jiang J Nilsson-Ehle P Xu N. Influence of liver cancer on lipid and lipoprotein metabolism. Lipids Health Dis. (2006) 5:4. 10.1186/1476-511X-5-4 16515689 PMC1420303

[43] Malaguarnera M Giugno I Trovato B Panebianco M Restuccia N Ruello P. Lipoprotein(a) in cirrhosis. a new index of liver functions? Curr Med Res Opin. (1996) 13:479–85. 10.1185/03007999609115228 9010614

[44] Basili S Andreozzi P Vieri M Maurelli M Cara D Cordova C. Lipoprotein (a) serum levels in patients with hepatocarcinoma. Clin Chim Acta. (1997) 262:53–60. 10.1016/S0009-8981(97)06533-9 9204209

[45] Rosas S Joffe M Wolfe M Brayman K Rader D. Effects of renal replacement therapy on plasma lipoprotein(a) levels. Am J Nephrol. (2008) 28:361–5. 10.1159/000112225 18057868 PMC2786011

[46] Kronenberg F Trenkwalder E Lingenhel A Friedrich G Lhotta K Schober M. Renovascular arteriovenous differences in Lp[a] plasma concentrations suggest removal of Lp[a] from the renal circulation. J Lipid Res. (1997) 38:1755–63. 10.1016/S0022-2275(20)37150-9 9323585

[47] Kronenberg F. Causes and consequences of lipoprotein(a) abnormalities in kidney disease. Clin Exp Nephrol. (2014) 18:234–7. 10.1007/s10157-013-0875-8 24129557

[48] Lange K Nave A Liman T Grittner U Endres M Ebinger M. Lipoprotein(a) levels and recurrent vascular events after first ischemic stroke. Stroke. (2017) 48:36–42. 10.1161/STROKEAHA.116.014436 27856951

[49] Hong X Wu D Lu J Zheng Y Tu W Yan J. Lipoprotein (a) as a predictor of early stroke recurrence in acute ischemic stroke. Mol Neurobiol. (2018) 55:718–26. 10.1007/s12035-016-0346-9 28004340

[50] Arnold M Schweizer J Nakas C Schütz V Westphal L Inauen C. Lipoprotein(a) is associated with large artery atherosclerosis stroke aetiology and stroke recurrence among patients below the age of 60 years: results from the BIOSIGNAL study. Eur Heart J. (2021) 42:2186–96. 10.1093/eurheartj/ehab081 33709115

[51] Nestel P Barnes E Tonkin A Simes J Fournier M White H. Plasma lipoprotein(a) concentration predicts future coronary and cardiovascular events in patients with stable coronary heart disease. Arterioscler Thromb Vasc Biol. (2013) 33:2902–8. 10.1161/ATVBAHA.113.302479 24092750

[52] Burgess S Ference B Staley J Freitag D Mason A Nielsen S. Association of LPA variants with risk of coronary disease and the implications for lipoprotein(a)-lowering therapies: a mendelian randomization analysis. JAMA Cardiol. (2018) 3:619–27. 10.1001/jamacardio.2018.1470 29926099 PMC6481553

[53] Moriarty P Gray J Gorby L. Lipoprotein apheresis for lipoprotein(a) and cardiovascular disease. J Clin Lipidol. (2019) 13:894–900. 10.1016/j.jacl.2019.09.010 31753721

[54] Nurmohamed N Kraaijenhof J Stroes E. Lp(a): a new pathway to target? Curr Atheroscler Rep. (2022). 10.1007/s11883-022-01060-4 36066785 PMC9534805

[55] Madsen C Kamstrup P Langsted A Varbo A Nordestgaard B. Lipoprotein(a)-lowering by 50 mg/dL (105 nmol/L) may be needed to reduce cardiovascular disease 20% in secondary prevention: a population-based study. Arterioscler Thromb Vasc Biol. (2020) 40:255–66. 10.1161/ATVBAHA.119.312951 31578080

[56] O'Donoghue M Fazio S Giugliano R Stroes E Kanevsky E Gouni-Berthold I. Lipoprotein(a), PCSK9 inhibition, and cardiovascular risk. Circulation. (2019) 139:1483–92. 10.1161/CIRCULATIONAHA.118.037184 30586750

[57] Ference B. The potential clinical benefit of lowering lipoprotein(a). JAMA. (2022) 327:1653–5. 10.1001/jama.2022.5333 35368050

